# Identification of radiation responsive RBC membrane associated proteins (RMAPs) in whole-body γ-irradiated New Zealand white rabbits

**DOI:** 10.1016/j.btre.2023.e00783

**Published:** 2023-01-18

**Authors:** Jubilee Purkayastha, Priyanka Grover, Prabuddho Mukherjee, Kamendra Kumar, Sudhir Chandna

**Affiliations:** a Department of Molecular and Radiation Biosciences (MARB), Institute of Nuclear Medicine and Allied Sciences (INMAS), Defence Research and Development Organisation (DRDO), Brig SK Majumdar Marg, Timarpur, Delhi 110054, India; b Department of Oncology, Lombardi Comprehensive Cancer Center, Georgetown University Medical Center, Washington-DC, United States

**Keywords:** RBC membrane, RRPs, RMAPs, Biomarker, Radiation biodosimeter

## Abstract

This study is aimed to identify radiation-responsive RBC Membrane Associated Proteins (RMAPs) in Rabbits *in vivo*. Male New Zealand White rabbits were exposed to a single acute total body γ-radiation dose of 2 Gy at a dose rate of 0.746 Gy/min. Following this, at early time points of 6 h till the 7 d, RMAPs were collected and analyzed by MALDI-TOF-MS. Bioinformatics analysis was conducted to explore the biological functions of these proteins. Based on fold change, radiation responsiveness, GO, pathway enrichment, and hub position in the PPI network, we identified seven RMAPs as potential biomarker candidates viz., PVALB, PRKCB, GPD1, CP2G1, CSNK2B, ATP1B1, TPI1. As per KEGG enrichment, most of the proteins were implicated in cellular radiation response, oxidative damage, DNA repair, apoptosis, immune response, and cell signaling. This study forms the foundation for RMAPs-based Proteomic strategies for high throughput radiation bio-dosimetry for triage in the case of a radiological/nuclear incident.

## Highlights

• Rapid triage through biological dosimetry assays is an utmost necessity for handling of major radiation emergencies.
• This study is aimed to identify radiation-responsive RBC membrane Associated Proteins (RMAPs) in Rabbit *in vivo*.
• We have identified differentially expressed proteins in RBC ghosts/membranes at early as well as late time-points (6 h up to 7 days) following whole-body γ-irradiation of rabbits at a clinically relevant dose of 2 Gy.
• The results of the present work lay a scientific foundation for the first time towards developing RMAPs based Proteomic strategies for high throughput radiation bio-dosimetry for triage.

**Abbreviations**
RBC, red blood cell
RRPs, radiation responsive proteins
RMAPs, RBC membrane associated proteins, TBI, Total body irradiation
MALDI, matrix-assisted laser desorption/ionization
TOF, time of flight
MS, mass spectrometry
GO, gene ontology
KEGG, kyoto encyclopedia of genes and genomes
VIP, variable importance in projection
PBS, phosphate-buffered saline
EDTA, ethylenediamine tetra acetic acid
PMSF, phenylmethylsulfonyl fluoride
BSA, bovine serum albumin
CHAPS, 3-[(3-cholamidopropyl) dimethylammonio]-1-propane sulfonate
SDS, sodium dodecyl sulfate
DTT, dithiothreitol
PRKCB, protein kinase C beta
ATP1B1, sodium/potassium-transporting ATPase subunit beta-1, CYP2G1, Cytochrome P450 2G1
GPD1, glycerol-3-phosphate dehydrogenase [NAD(+)], cytoplasmic, CSNK2B, casein kinase II subunit beta
TPI1, triosephosphate isomerase
PVALB, parvalbumin alpha

## 1 Introduction

Rapid triage is an utmost necessity for handling of major radiation emergencies and for providing timely medical intervention [1,2]. Besides assessing clinical symptoms, hematopoietic investigations are used as first-line supportive tests. However, these are insufficient for assessing absorbed radiation doses. Biological dosimetry assays and biomarkers can greatly help in assessing absorbed doses, especially during large-scale incidents. While cytogenetic techniques can assess absorbed radiation doses precisely, these are time-consuming and may be inadequate for mass screening in the incident of a major nuclear or radiological event [3,4]. Therefore, efforts have been ongoing to develop multiple new biomarkers and assays for timely aid for rapid triage selection.

Following exposure to ionizing radiation, dose- and time-dependent alterations in the levels of specific transcripts or proteins/polypeptides are elicited in various tissues. Multiple intracellular and systemic signal transduction pathways are induced by radiation; thereby providing a variety of potential biomarkers and end-points [5,6]. These variations can be registered by different omics-based approaches [4], including detection of changes in protein profiles for estimation of absorbed radiation doses [7,3]. Studies on peripheral blood serum/plasma proteins have been particularly useful, and dose-dependent alterations in the expression of alpha-actinin-1 (ACTN1), Ferredoxin reductase (FDXR), DNA-binding protein 2 (DDB2), FMS-like tyrosine kinase 3 ligand (Flt-3 L), Serum Amyloid-α (SAA), matrix metalloproteinase 9 (MMP9), Fibrinogen-beta (FGB) and pentarexin-3 proteins have been detected in different experimental animals [2,8]. However, there is no study conducted on the effect of ionizing radiation on protein profile of erythrocytes, despite the abundance of this cell type in the blood. In the present study, we have identified differentially expressed proteins in RBC ghosts/membranes at early as well as late time-points (from 6 hour up to 7 day) following whole-body γ-irradiation of rabbits at a clinically relevant dose of 2 Gy. Bioinformatic analysis was used to identify functional associations/ enriched pathways of these candidate proteins. Identification of radiation-responsive proteins in peripheral blood with potential for assessing absorbed radiation doses within few hours or even up to few days post-exposure can be quite useful for the mass-screening of radiation-exposed individuals. This is the first report to our knowledge on Radiation Responsive RBC Membrane Associated Proteins (RMAPs) identified from the whole-body irradiated rabbits.

## 2 Materials and methods

### 2.1 Animals

Disease-free New Zealand White (NZW) male rabbits (8–12 months old; 2.0–2.5 kg body weight) obtained from Animal House, Institute of Nuclear Medicine and Allied Sciences (INMAS) were used for the experiments. Each rabbit was housed in an individual cage and sustained on a standard pellet diet as well as fresh vegetables, ad libitum water and a 12 h light/dark cycle.

### 2.2 Total body irradiation (TBI) and blood collection

A teletherapy unit (Bhabhatron II, Panacea Medical Technologies, India) with ^60^Co source was used for whole-body irradiation of rabbits. Each male New Zealand White rabbit individually received a single acute total body γ-radiation dose of 2 Gy at a dose rate of 0.746 Gy/min. The rabbits were confined using a standard pie jig, anesthetized with 120 mg/kg b.w. ketamine (i.p.) during the procedure. Untreated and irradiated animals were provided with standard dietary requirements based on pellets, fresh vegetables and *ad libitum* filtered water, and were kept discretely in cages under health monitoring throughout the experiment. Blood was collected from central ear vein at 6 h, 24 h, 48 h and 96 h as well as 7 d post-irradiation in K_2_EDTA blood collection tubes (BD Biosciences, San Jose, California, Cat no. 368,856). Unirradiated Rabbits served as control group.

### 2.3 Ethics statement

All experiments carried out on animals have been approved by the Institutional Animal Ethics Committee of the Institute of Nuclear Medicine and Allied Sciences (INMAS) (Institutional Animal Ethics Committee reference: INM/IAEC/2018/17Ext/Ext7 dated 18th June 2019).

### 2.4 RBC ghost preparation, protein extraction and quantification

The original methods of Schatzmann and Rossi [9] and Wolf [10] with slight modifications were used for RBC membranes (ghosts) preparation after removing platelets and white-blood cells. Briefly, collected blood sample after centrifugation at 600 g, plasma was separated out and blood cells were then treated with phosphate-buffered saline (PBS) and added to 3 ml ficoll histopaque (Sigma Aldrich, Histopaque®−1077) resulting in separation into three layers. Upper two layers (consisting of mainly granulocytes and platelets) were removed and the cells centrifuged for 3 min at 4°C, 3000 rpm; after washing with cold phosphate-buffered saline (PBS). The cells were then centrifuged at 13,000 rpm, at 4°C for 10 min after resuspending with lysis buffer [(5 mM, phosphate Buffer (pH=8), 1 mM, Ethylenediamine tetra acetic acid (EDTA), 1 mM, phenylmethylsulfonyl fluoride (PMSF)]. A Bradford protein assay kit (Bio-Rad, USA) was used to find out the total protein concentration based on Bradford's [11] methodology. Bovine serum albumin (BSA) was used as the standard.

### 2.5 Two-dimensional electrophoresis

Protein samples were dissolved in rehydration buffer (4% 3-[(3-Cholamidopropyl) dimethylammonio]−1-propane sulfonate (CHAPS), 7 M urea, 0.2% (v/v) Ampholyte 3–10, 2 M thiourea, 15 mM dithiothreitol, bromophenol blue); and were added at appropriate concentration to pH 4–7 immobilized pH gradient (IPG; Bio-Rad, USA, Cat no. 1,632,001 and 1,632,008) strips of 7 cm (12μg protein loaded) as well as 17 cm (75μg protein loaded) length, and rehydrated overnight. A Protean IEF cell (Bio-Rad, USA) was used to separate proteins in the first dimension with focusing programmed up to 30,000 volt hours (Vh) for 7 cm IPG strips (pH 4–7) or 50,000 Vh for 17 cm IPG strips (pH 4–7), respectively, and stored at −70 °C until second dimensional electrophoresis.

For second dimensional electrophoresis, strips were immersed for 10 min each in equilibration buffer-I (30% w/v glycerol, 1% w/v DTT in 50 mMTris/HCl buffer, 6 M urea, and 2% w/v SDS, pH 8.8) followed by buffer-II (30% w/v glycerol, 4% w/v iodo-acetamide in 50 mMTris/HCl buffer, 6 M urea and 2% w/v SDS, pH 8.8). Following equilibration, electrophoresis was conducted by the method of Blackshear [12] using 10% SDS-PAGE gel at 80 V for 4 h (for 7 cm strip) or at 50 V for 18 h (for the 17 cm strip). The gels were later silver stained as per standard method [13].

### 2.6 2DE gel imaging and spot analysis

2DE gel images were obtained using Epson Expression 12000XL Scanner (EPSON; Model J331B/EU235). Molecular weight and pI values of protein spots in 2DE gels were determined using PD Quest Software version 8.0 (Bio-Rad USA), and relative abundance as well as differential expression patterns were calculated. Alterations in the protein expression (fold changes) were calculated as the ratio of densitometric values obtained for a respective protein spot in the replicate gels when compared with that of the control gels.

### 2.7 Mass spectrometry analysis and mascot database searches for protein identification

With the use of thin-walled bottom-cut 200 μl PCR tubes, protein spots were carefully excised without contamination by neighbouring proteins or skin keratin. A matrix-assisted laser desorption/ionization time-of-flight mass spectrometer was used to analyze protein spots digested by trypsin (Sandor Proteomics Pvt Ltd, Hyderabad) using Bruker Daltonics UltraflexTM III Mass Spectrometer with internal calibration of spectra using trypsin auto-digestion products. The data obtained from MALDI-TOF-MS were further used to identify proteins using the MASCOT protein database search engine available at http://www.matrixscience.com. Search parameters were trypsin digestion with one missed cleavage, variable modifications (oxidation of methionine and carbamido-methylation of cysteine), and the peptide mass tolerance of ±70 ppm with +1 charge state. Swissprot and NCBInr databases were searched with *Oryctolagus cuniculus* as the preferred taxonomy. Positively identified proteins were those with probability-based MOWSE scores exceeding their thresholds (P 0.05).

### 2.8 Protein functional classification, hierarchical clustering, pathway, PPI network, multivariate and correlation analysis

The Venn diagram of the radiation responsive proteins (RRPs), which are RBC membrane associated at different time-points, was constructed via the Draw Venn Diagram website (https://dashboard.visme.co/v2/projects/own). GO enrichment analysis and Kyoto Encyclopedia of Genes and Genomes (KEGG) pathway analyses of the differentially expressed RRPs were performed to map respective genes to the possible molecular pathways using online package http://bioinformatics.sdstate.edu/go/ and http://bioinformatics.sdstate.edu/go61/, respectively. For statistical significance study, the threshold of false discovery rate (FDR) <0.05 was set as the standard and FDR is calculated based on nominal P-value from the hypergeometric test. Heat Map analysis of the differentially expressed Radiation Responsive RBC Membrane Associated Proteins (RMAPs) was performed using online package https://software.broadinstitute.org/morpheus/. In order to further determine protein-protein interaction networks and to gain valuable biological functions, interactions amongst the proteins were analysed with inBio Discover™ online (https://inbiodiscover.com/#login) that builds upon the high coverage, high quality protein-protein interaction network inBio Map™. MetaboAnalyst 5.0 software was used for the correlation and multivariate analysis (www. metaboanalyst.ca). To analyze the data, row-wise normalization was performed in order to obtain Gaussian distributions. They were then normalized log10 for analysis.

### 2.9 Statistical analysis

For differential study, results from four replicate gels of RBC Membrane Proteins at each time points were computationally pooled with PD Quest (Bio-Rad, USA) software and matched spots were compared with that of Control. To ensure consensus of the differentially expressed spots among replicates and to remove any possible artefact, the spots were manually curated. A paired *t*-test analysis was conducted to determine whether the relative change in protein expression between the ‘irradiated sample’ and the ‘control sample’ was statistically significant. The mean values of normalized spot intensities from duplicate analytical gels from four replicates (two biological with two analytical replicates each) were analyzed.

## 3 Results

### 3.1 Radiation responsive RBC membrane associated proteins (RMAPs) of whole-body irradiated rabbits

**Fig. 1:**
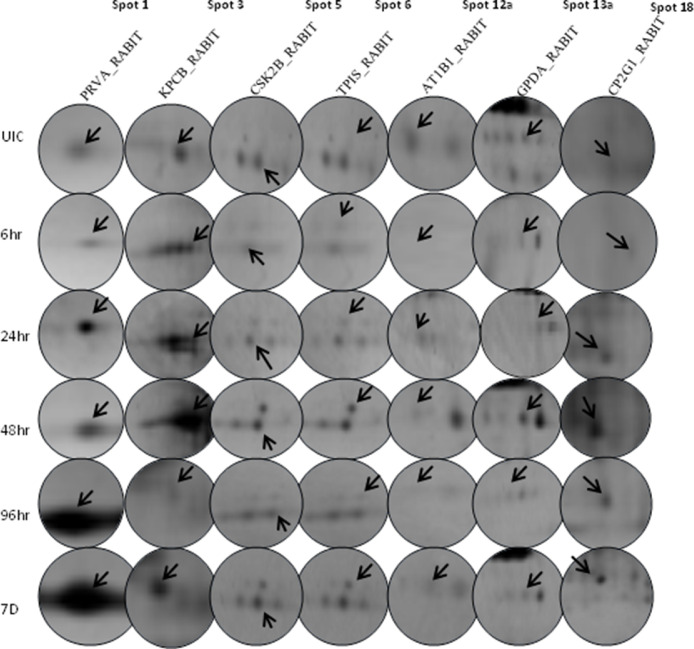
Representative 2-DE spots of differentially expressed proteins in Whole Body γ-irradiated New Zealand White Rabbits under 2 Gy irradiation at different Time Points. UIC-Un-irridated Control, 6 hr: at 6 hr after irradiation, 24 hr: at 24 hr after irradiation, 48 hr: at 48 hr after irradiation, 96 hr: at 96hr after irradiation, 7D: On 7th Day after irradiation. Total proteins were separated by 2-DE (IPG strips pH 4–7, 7 cm and 17 cm) and silver stained. Spot detection and image analysis by PD Quest™ software (BioRad).

**Fig. 2:**
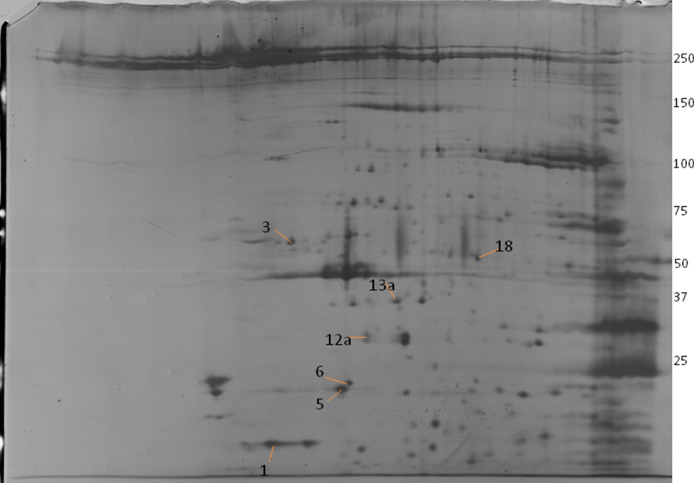
A representative 2-DE Maxi gel of RBC Membrane Associated Proteins (RMAPs) from Whole Body γ-irradiated New Zealand White Rabbits used for Spot Excision and Identification. Proteins were separated in the first dimension by a pH 4 to 7 immobilized pH gradient gel (length, 17 cm) and then in the second dimension by a 10% polyacrylamide gel. Spots were excised, and the corresponding proteins were identified by MALDI-TOF-MS and database searches. The spots are labelled on the gel according to the numbers presented in Table 1.

Two-dimensional electrophoresis provided first-hand indication of radiation-induced alterations in the expression levels of RMAPs at early (6 h) as well as up to late (7 d) time-points (Fig. S1). Densitometric analysis further revealed several differentially expressed spots with intensities varying up to ∼8 fold higher (e.g., Fig. 1, Table 1) and ∞ fold lower (spot disappears) expression. Molecular weight and pI values were calculated with the PD Quest Software version 8.0 (Bio-Rad, USA), and most of the experimental values of these differentially expressed protein spots coordinated well with theoretical values (Fig. 2; Table 2). Minor differences between experimental and theoretical masses and/or pI values are likely to be caused by post-translational modifications such as phosphorylation of multiple residues, proteolytic processing or the cleavage of alkaline regions.

**Table 1:** Differential RBC Membrane Associated Protein (RMAPs) Spots identified from Whole Body γ-irradiated New Zealand White Rabbits (Radiation Dose: 2 Gy). 6 hr: at 6 hr after irradiation, 24 hr: at 24 hr after irradiation, 48 hr: at 48 hr after irradiation, 96 hr: at 96hr after irradiation, 7D: On 7th Day after irradiation.6 hr: at 6 hr after irradiation, 24 hr: at 24 hr after irradiation, 48 hr: at 48 hr after irradiation, 96 hr: at 96hr after irradiation, 7D: On 7th Day after irradiation.

Spot No.	Protein Identity	6hr			24hr			48hr			96hr			7D		
		Fold Change ± SE	*p*-value	log2Fold Change	Fold Change ± SE	*p*-value	log2Fold Change	Fold Change ± SE	*p*-value	log2Fold Change	Fold Change ± SE	*p*-value	log2Fold Change	Fold Change ± SE	*p*-value	log2Fold Change
1	Parvalbumin alpha OS=Oryctolagus cuniculus OX=9986 GN=PVALB PE=1 SV=2 (ID: PRVA_RABIT; AC: P02624)	0.65 ± 0.46	1.04E-03	−0.6027596	2.11 ± 2.1	3.36E-03	1.078951	2.19 ± 1.33	6.75E-04	1.135863	7.5 ± 3.65	6.52E-05	2.906891	8.20 ± 2.66	1.57E-05	3.035624
3	Protein kinase C beta type OS=Oryctolagus cuniculus OX=9986 GN=PRKCB PE=2 SV=3 (ID: KPCB_RABIT, AC: P05772; P05773;)	0.50 ± 0.62	3.72E-03	−0.986074	0.504 ± 0.93	4.83E-03	−0.98607	3.90 ± 4.45	2.23E-03	1.963969	0.752 ± 0.38	8.85E-03	−0.41067	2.31 ± 2.06	1.74E-03	1.213695
5	Casein kinase II subunit beta OS=Oryctolagus cuniculus OX=9986 GN=CSNK2B PE=2 SV=1(ID: CSK2B_RABIT, AC: P67873; P07312; P13862)	0.50 ± 0.06	1.23E-02	−0.997421	0.39 ± 0.08	1.24E-03	−1.34088	2.92 ± 0.73	6.45E-03	1.550151	0.12 ± 0.05	1.44E-03	−3.03376	2.79 ± 0.23	8.49E-04	1.482098
6	Triosephosphate isomerase OS=Oryctolagus cuniculus OX=9986 GN=TPI1 PE=1SV=1(ID: TPIS_RABIT, AC: P00939)	2.59 ± 0.41	7.21E-04	1.37771	2.02 ± 0.27	8.28E-04	1.015814	2.75 ± 0.22	2.83E-05	1.462269	2.06 ± 0.25	4.70E-04	1.045961	1.79 ± 0.84	2.93E-02	0.840635
12a.	Sodium/potassium-transporting ATPase subunit beta-1 OS=Oryctolagus cuniculusOX=9986 GN=ATP1B1 PE=1 SV=1(ID: AT1B1_RABIT, AC: Q9TT37)	∞	1.22E-03	0	2.19 ± 0.45	1.27E-03	1.132577	1.99 ± 0.84	6.00E-03	0.99458	∞	1.22E-03	0	2.22 ± 2.25	7.66E-03	1.153805
13a.	Glycerol-3-phosphate dehydrogenase [NAD(+)], cytoplasmic OS=Oryctolaguscuniculus OX=9986 GN=GPD1 PE=3 SV=2(ID: GPDA_RABIT, AC: P08507)	0.62 ± 0.46	1.57E-02	−0.675501	1.29 ± 0.59	2.91E-02	0.377717	3.20 ± 2.27	4.33E-03	1.678404	0.70 ± 0.34	4.17E-03	−0.49878	0.62 ± 0.59	3.37E-02	−0.67652
18	Cytochrome P450 2G1 OS=Oryctolagus cuniculus OX=9986 GN=CYP2G1 PE=1 SV=1(ID: CP2G1_RABIT, AC: P24461)	0.50 ± 0.07	2.79E-02	−0.993890	4.49 ± 0.75	9.72E-04	2.168037	5.89 ± 0.36	1.62E-05	2.559018	4.46 ± 0.67	6.95E-04	2.230039	4.69 ± 0.78	3.28E-04	2.158241

**Table 2:** Differential RBC Membrane Associated Protein (RMAPs) Spots identified from Whole Body γ-Irradiated New Zealand White  Rabbits (Radiation Dose: 2 Gy). The table reports: 1) Mr/pI (Observed/ Theoretical) 2) pI Range (http://isoelectricpointdb.org/) 3) Relative Abundance (%), 4) the peptides generated by trypsin digestion, the platform for their identification by mass spectrometry.

Spot No.	Protein Identity (Accession No.)	Observed Mr/pI	Theoretical Mr/pI	pI Range	Relative Abundance(%)	Matched Peptides
1	Parvalbumin alpha OS=Oryctolagus cuniculus OX=9986 GN=PVALB PE=1 SV=2 (AC: P02624)	12.0/5.1	12.057/5.05	4.4–5.1	92.50	-.MAMTELLNAEDIKK.A -.MAMTELLNAEDIKK.*A* + 2 Oxidation (M) M.AMTELLNAEDIKK.A K.AIGAFAAAESFDHK.K
3	Protein kinase C beta type OS=Oryctolagus cuniculus OX=9986 GN=PRKCB PE=2 SV=3 (AC: P05772; P05773;)	71.2 /5.2	77.918/6.57	4.2–7.0	87.98	K.ESDKDR.R K.GSFGKVMLSER.*K* + Oxidation (M) K.LDNVMLDSEGHIK.I K.EAVAICKGLMTK.H K.RDTSNFDK.E
5	Casein kinase II subunit beta OS=Oryctolagus cuniculus OX=9986 GN=CSNK2B PE=2 SV=1 (AC: P67873; P07312; P13862)	22.8/5.4	25.268/5.33	2.7–5.5	88.67	K.FNLTGLNEQVPHYR.Q R.GIAQMLEK.*Y* + Oxidation (M) K.YQQGDFGYCPR.V K.RPANQFVPRLYGFK.I R.LYGFKIHPMAYQLQLQAASNFK.*S* + Oxidation (M)
6	Triosephosphate isomerase OS=Oryctolagus cuniculus OX=9986 GN=TPI1 PE=1 SV=1 (AC: P00939)	24.6/5.5	26.894/7.10	4.5–8.0	83.61	R.KFFVGGNWK.M K.NLGELITTLNAAK.V R.RHVFGESDELIGQK.V R.HVFGESDELIGQK.V K.SNVSDAVAQSTR.I
12a	Sodium/potassium-transporting ATPase subunit beta-1 OS=Oryctolagus cuniculus OX=9986 GN=ATP1B1 PE=1 SV=1 (AC: Q9TT37)	34.4/5.6	35.317/8.61	4.2–8.8	73.79	R.VAPPGLTQVPQIQK.T R.VAPPGLTQVPQIQKTEIAFRPSDPK.S K.SYEEYVVNIVR.F K.DDMVFEDCGDVPSEPKER.*G* + Oxidation (M) K.LNRVLGFKPKPPK.N R.VLGFKPKPPKNDSLEFSPGTK.Y
13a	Glycerol-3-phosphate dehydrogenase [NAD(+)], cytoplasmic OS=Oryctolagus cuniculus OX=9986 GN=GPD1 PE=3 SV=2 (AC: P08507)	36.8/5.8	38.213/6.32	4.3–7.9	84.83	R.VTMWVFEEDIGGKK.L K.GHLKANAIGISLIK.G K.FCETTIGCKDQAQGQLLK.Q K.DQAQGQLLKQLMQTPNFR.I K.AAVIRLGLMEMIAFAK.*L* + 2 Oxidation (M) R.LGLMEMIAFAK.L K.EMLNGQKLQGPETAR.*E* + Oxidation (M)
18	Cytochrome P450 2G1 OS=Oryctolagus cuniculus OX=9986 GN=CYP2G1 PE=1 SV=1 (AC: P24461)	55.2/6.2	56.992/8.07	4.4–8.2	83.33	K.YGPVFTVYMGPRPVVILCGHEAVK.*E* + Oxidation (M) R.SIEERIQEEAGYLLEEFR.K K.GAPIDPTFFLSR.T R.MINESFIEMSTPWAQLYDMYSGVMQYLPGR.H + 4 Oxidation (M) K.VNEASLDPQNPR.D R.YGFLLIMKHPEVQTK.I R.IPSVDDRVK.M

Among the identified Radiation Responsive RBC Membrane Associated Proteins (RMAPs) (Fig. 1, Table 1), Protein kinase C beta (PRKCB) (KPCB_RABIT) wrt control was down-regulated at 6 h and 24 h, showed upregulation at 48 h, downregulated at 96 h and upregulated again on 7d. This type cyclic regulation of expression of this protein might be due to internal physiological changes which are explained for all the RRPs in this study under ‘Time kinetics pattern of the identified radiation responsive RMAPs’.

Erythrocyte membranes contain multiple protein kinases as reported earlier. Human and rabbit erythrocyte membranes contain a protein kinase that phosphorylates membrane proteins using ATP as its phosphoryl donor, as well as another kinase that can either phosphorylate membrane proteins using ATP or GTP [14]. RBC membrane phospholipid scrambling is mediated by PKC [15] and is reported to be involved in H3T6 phosphorylation [16].

Sodium/potassium-transporting ATPase subunit beta-1 (ATP1B1) (AT1B1_RABIT) also showed early response and disappeared at 6 h as well as 96 h. (Fig. 1 and Table 1).

There are several factors involved in the maintenance of RBC deformability, which is one of the very important characteristics of RBC that allows them to pass through narrow capillaries while in circulation. An example is the Na,K-ATPase, known as a crucial enzyme in maintaining intracellular ionic homeostasis, thereby affecting cellular volume and, subsequently, RBC deformability. Na,K-ATPase in RBCs controls RBC deformability, and changes in Na,K-ATPase activity are commonly accompanied by changes in RBC deformability [17].

Cytochrome P450 2G1 (CYP2G1) (CP2G1_RABIT) was found to be significantly downregulated at the earliest time-point of 6 h wrt control and then was upregulated in later time points (Fig. 1 and Table 1). Cytochrome P450 enzymes (CYPs or P450s) are of immense importance as their activity can cause oxidative stress [18]. The correlation Between Red Blood Cell Survival and Cytochrome P450 1A2 Enzyme Activity was studied by Dumont et al. 2013 [19] and Cytochrome P450-dependent toxicity of primaquine and dapsone were found in human erythrocytes [20,21].

On the other hand, Glycerol-3-phosphate dehydrogenase [NAD(+)], cytoplasmic (GPD1) (GPDA_RABIT) was downregulated in majority of the time-points tested, except 24 h and 48 h where it was up regulated (Fig. 1 and Table 1). In RBC, glycolytic enzymes were found to be associated with the band 3-Ank1 complex, and both open and closed forms of ankyrin were capable of accommodating glyceraldehyde-3-phosphate dehydrogenase [22]. The immunofluorescence and binding assays of Rodalski et al. 1989 [23] demonstrated that glycerol-3-phosphate dehydrogenase is associated with the red cell membrane. In addition to rapid adjustment of the ratio of cell volume to surface area, presence of energy-producing enzymes on the RBC membrane may facilitate cytoskeletal protein conformational changes as well as dynamic morphological changes of RBCs that facilitate movement through tissues and microvasculature [22].

Casein kinase II subunit beta (CSNK2B) (CSK2B_RABIT) was down regulated at 6 h 24 h, and 96 h; while it showed upregulation at 48 h and on 7 d (Fig. 1 and Table 1). Casein kinase is a well-conserved protein kinase implicated in cell metabolism and differentiation [24,25].

Earlier, multiple forms of Casein Kinases had been identified from rabbit red blood cells, although, the function of these protein kinases in rabbit red blood cells is still not very clear. It was suggested that a modification of certain ribosomal proteins in the reticulocyte system may contribute to the regulation of ribosome activity through these enzymes. In addition, these enzymes were assigned to perform some other unknown function in matured erythrocytes [26].

The other prominently abundant over-expressed protein following γ-irradiation at 2 Gy was Triosephosphate isomerase (TPI1) (TPIS_RABIT) which also showed early response and was up regulated at all the time points starting with 6 h post-irradiation (Fig. 1 and  Table 1).

To maintain proper ion concentrations and appropriate surface area/volume ratios, RBCs require energy to sustain their ability to change morphology without breaking [27,28]. The major source of energy production in RBCs is *via* the glycolysis pathway [29], producing methylglyoxal as byproduct, which requires to be detoxified. A novel interaction between triosephosphate isomerase (TPI1) and Parkinsonism-associated protein (DJ-1/Park7) has been found that is relevant to methylglyoxal detoxification, and a physical link has been established between RBC energy production and by product detoxification [22].

The erythrocyte TPI is known to play significant role in the glycolytic metabolism as well as pathogenesis in certain disease conditions such as well-established red cell enzymopathies [30,31]. The implications of radiation-induced alterations in its expression in our study are discussed later.

Amongst the differentially expressed spots identified, the Parvalbumin alpha (PVALB) (PRVA_RABIT) protein was initially down regulated by 6 h but showed significant up regulation at 96 h onwards (Fig. 1 and Table 1).

Parvalbumin is a small, stable protein containing EF-hand type calcium binding sites with low molecular weight (typically 9–11 kDa) and is involved in calcium signaling [32] and many other physiological processes [33]. Detection of this protein in RBC ghosts is rather unexpected, since its presence in erythrocytes is not yet clearly established. While its most abundant calcium binding sibling ‘calmodulin’ has ubiquitous presence in the mammalian tissues [34], the calcium binding role of parvalbumin is primarily reported in myocytes and certain other cell types [35] as discussed later.

### 3.2 Time kinetics pattern of the identified radiation responsive RMAPs

**Fig. 3:**
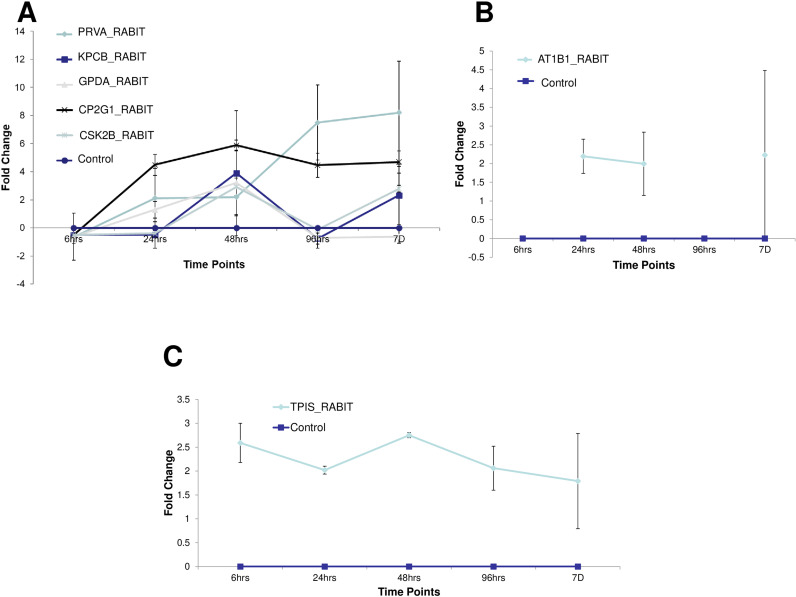
Time kinetics for radiation responsive RMAPs measured as fold change w.r.t Control using PD Quest software. Each data point, mean ± SE values of two independent experiments of two biological replicate samples having two technical replicates from each experiments. (A) Proteins Down regulated at 6hr post irradiation, (B) Proteins Disappeared at 6hr and 96 hr post irradiation (C) Protein Upregulated throughout all time points post irradiation.

The time kinetics (6h up to 7d) of post-irradiation protein expression changes of all protein spots is depicted in Fig. 3. The Protein Expression pattern was divided in to three groups based on time kinetics viz., (a) Proteins Down regulated at 6h post irradiation, (b) Proteins Disappeared at 6h and 96 h post irradiation (c) Protein Upregulated throughout all time points post irradiation. PRVA_RABIT, KPCB_RABIT, GPDA_RABIT, CP2G1_RABIT, CSK2B_RABIT are the proteins under first category; AT1B1_RABIT is the protein under second category and TPIS_RABIT is the protein under third category. There is another category that emerge from Fig. 3 is where the expression pattern of the protein are following a cyclic fashion of up and down regulation. KPCB_RABIT, AT1B1_RABIT, GPDA_RABIT, CSK2B_RABIT are the protein under Cyclic Expression Pattern category.

Transcription factors may have been activated by signals from the exposure to radiation and might have affected protein synthesis by altering transcription. This might have resulted in higher/lower production of a particular protein. Indeed, both decreased and increased level of expression of these proteins may be due to physiological instability due to radiation exposure and cyclic expression may be a dynamic one as a mix of up-regulation and down-regulation for these RRPs to meet various cellular needs.

### 3.3 Homology of the identified radiation responsive RMAPs

A homology study of the identified proteins (Table S1) revealed homologues of proteins from *Mus musculus, Homo sapiens, Otolemur garnettii, Callithrix jacchus, Ictidomys tridecemlineatus, Rhinopithecus bieti, Ochotona princeps etc.* Thus, the proteins identified from *Oryctolagus cuniculus* in the present study emerged to be moderately to highly conserved among these different species following the blastP search and considering top three homologous species (84.55 to 100% amino acid identity).

### 3.4 Gene ontology, enriched functional categories, KEGG pathways and hierarchial clustering of the identified radiation responsive RMAPs

**Fig. 4:**
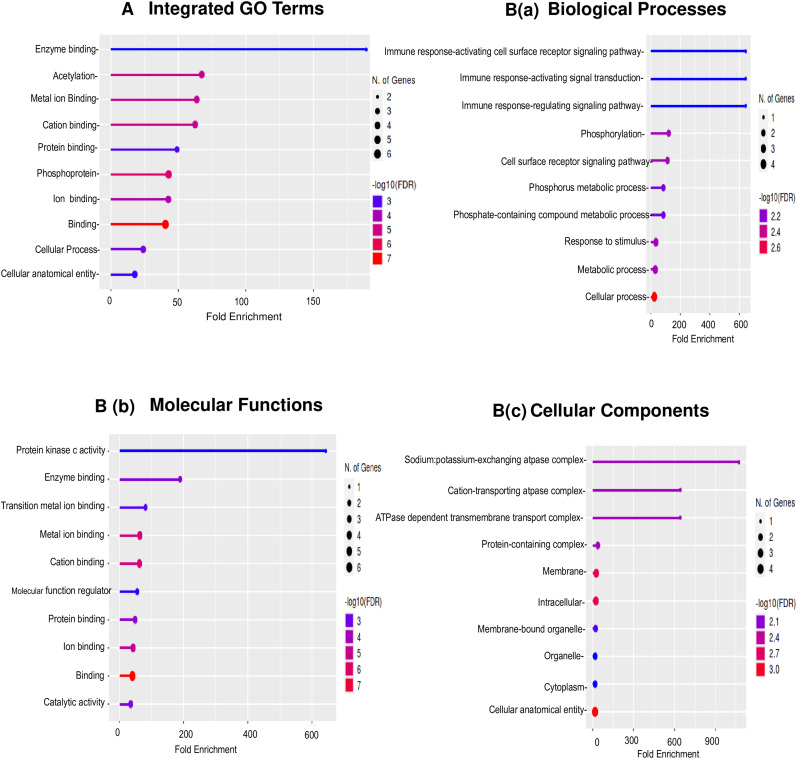
(A) Top 10 integrated results of gene ontology enrichment analyses using all candidate RRPs. (B) Comparative analysis of representative top 10 enriched categories for the Gene Ontology. B(a) Biological processes GO terms, B(b) Molecular function GO terms, B(c) Cellular Components GO terms. Significance was determined at P-value cut off (FDR) 0.05. The horizontal axis is the Fold Enrichment GO category, the left vertical axis is the GO Functional Category and the right vertical axis is the –log FDR of these GO Categories.

**Fig. 5:**
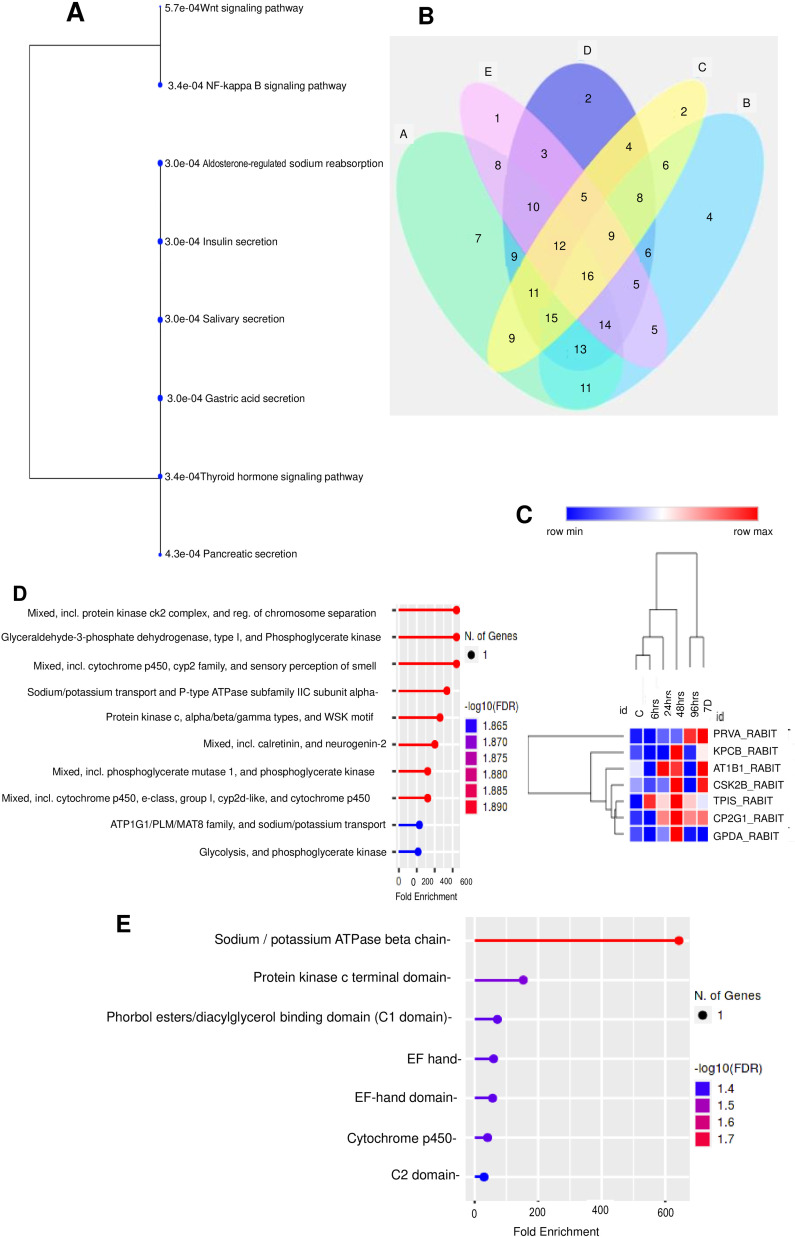
(A). KEGG hierarchical clustering tree summarizing the correlation among significant pathways listed in the Enrichment tab. Pathways with many shared genes are clustered together. Significance was determined at P-value cut off (FDR) 0.05. (B) Venn Diagram of the nos of time points of the expression of RRPs wrt fold change. A: >2 Fold Change, B: >3 Fold Change, C: >4 Fold Change, D: >5 Fold Change, E: >8 Fold Change. Numbers in the overlapping regions refer to the nos of time points of the RRPs, that were covered in more than one fold change points. (C) The heat map of 7 radiation responsive RMAPs at 6 hr, 24 hr, 48 hr, 96 hr and on day 7 post irradiation. Diagram presents the result of a two-way hierarchical clustering of 7 differentially expressed radiation responsive RMAPs and time points. (D) Enriched Local Protein Network Cluster (STRING) of the radiation responsive RMAPs. (E) Enriched Categories of Protein Family (Pfam) domains of the radiation responsive RMAPs. (D & E: Significance was determined at P-value cut off (FDR) 0.05. The horizontal axis is the Fold Enrichment GO category, the left vertical axis is the GO Functional Category and the right vertical axis is the –log FDR of these GO Categories).

To further understand the intracellular and extracellular functional associations of these candidate proteins, we performed gene ontology (GO) and KEGG Pathways analysis by using online package http://bioinformatics.sdstate.edu/go/ and http://bioinformatics.sdstate.edu/go61/, respectively. The majority of these proteins were drastically represented in three main GO categories: ‘biological process’, ‘molecular function’ and ‘cellular component’ (Fig. 4). The top 10 integrated GO results were visualized and are listed in Fig. 4(A). As shown in Fig. 4B(a) and Table S2, the top five GO Biological processes were “Immune response-activating cell surface receptor signalingpathway,” “Immune response-activating signal transduction,” “Immune response-regulating signalingpathway,” “Phosphorylation,” and “Cell surface receptor signalingpathway.” The top five GO Molecular functions as in Fig. 4B(b) were “Protein kinaseC activity,” “Enzyme binding,” “Transition metal ion binding,” “Metal ion binding,” and “Cationbinding.”The top five GO Cellular Components as in Fig. 4B(c) were were “Sodium:potassium-exchanging atpasecomplex,” “Cation-transporting atpasecomplex,” “ATPasedependent transmembranetransport complex,” “Protein-containing complex,” and “Membrane.” Kyoto Encyclopedia of Genes and Genomes (KEGG) pathway analyses of the Radiation Responsive RBC Membrane Associated Proteins (RMAPs) of whole-body irradiated rabbits revealed Wnt signaling, NF-kappa B signaling pathway, Aldosterone-regulated sodium reabsorption, Insulin secretion etc. as the top enriched signaling pathways (Fig. 5A). A Venn Diagram of the differentially expressed RRPs associated with RBC Membrane of TBI Rabbits at different time points, 6 h; 24 h, 48 h, 96 h, and 7d are shown in Fig. 5B. Heat maps are a commonly used visualization tool for proteomic data where the relative abundance of proteins is represented with color intensity. Fig. 5C shows a heatmap of all RRPs hierarchical clustering using the complete-linkage method together with the Euclidean distance. Heat Map showed that Proteins viz., PRVA_RABIT, CP2G1_RABIT, CSK2B_RABIT, AT1B1_RABIT under 7d and CP2G1_RABIT, KPCB_RABIT, CSK2B_RABIT, AT1B1_RABIT, TPIS_RABIT, GPDA_RABIT under 48 h were showing upregulated expression pattern whereas KPCB_RABIT, CSK2B_RABIT, AT1B1_RABIT under 96 h were mostly downregulated. As clearly shown in the Heat Map, all the proteins were down regulated at 6h except TPIS. From blue (low expression) through white to red (high expression), the expression of protein fold changes is colored according to a color scale. An illustration of degrees of relatedness between RRPs expression profiles is depicted in the dendrogram on the left hand side of the heat map. The dendrogram above the heat map is indicating relationships between the expression profiles of the RRPs across all of the samples evaluated during this study. Enriched local protein network cluster (STRING) and enriched categories of Protein Family (Pfam) domains of the radiation responsive RMAPs are shown in Fig. 5D & E.

### 3.5 Protein interaction of the identified radiation responsive RMAPs

**Fig. 6:**
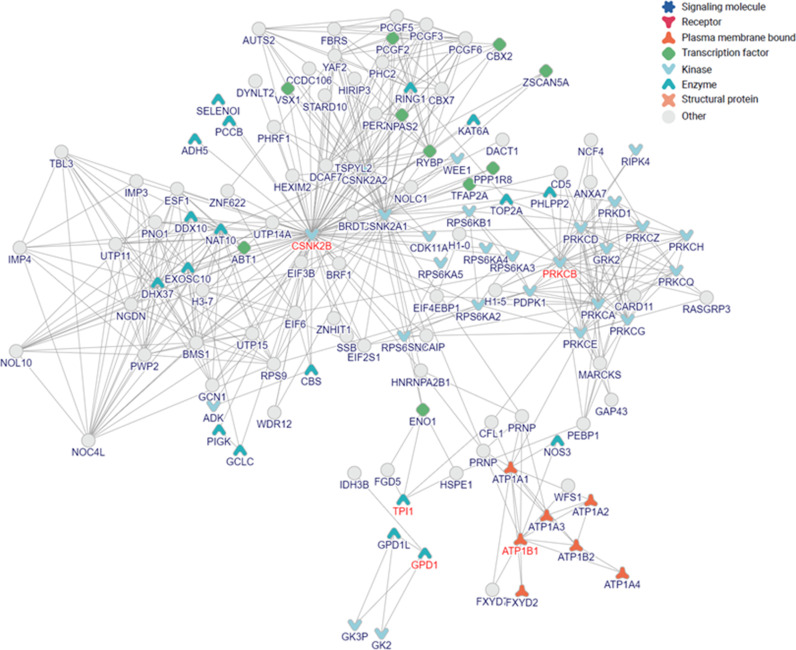
Protein Protein Interaction (PPI) networks of the identified key Radiation Responsive RBC Membrane Associated Proteins (RMAPs) with inBio Discover™.

With inBio Discover™, we could explore the interactions of the proteins in our study. We used the seven Radiation Responsive RBC Membrane Associated Proteins (RMAPs) to construct the networks and the PPI analysis was able to construct interaction networks using 5 of the identified RRPs. The inBio Discover™ results demonstrated that CSNK2B, PRKCB, ATP1B1, TPI1 emerged as the top four hub proteins from our study that was represented in the network (Fig. 6).

### 3.6 Multivariate and correlation analysis of the identified radiation responsive RMAPs

**Fig. 7:**
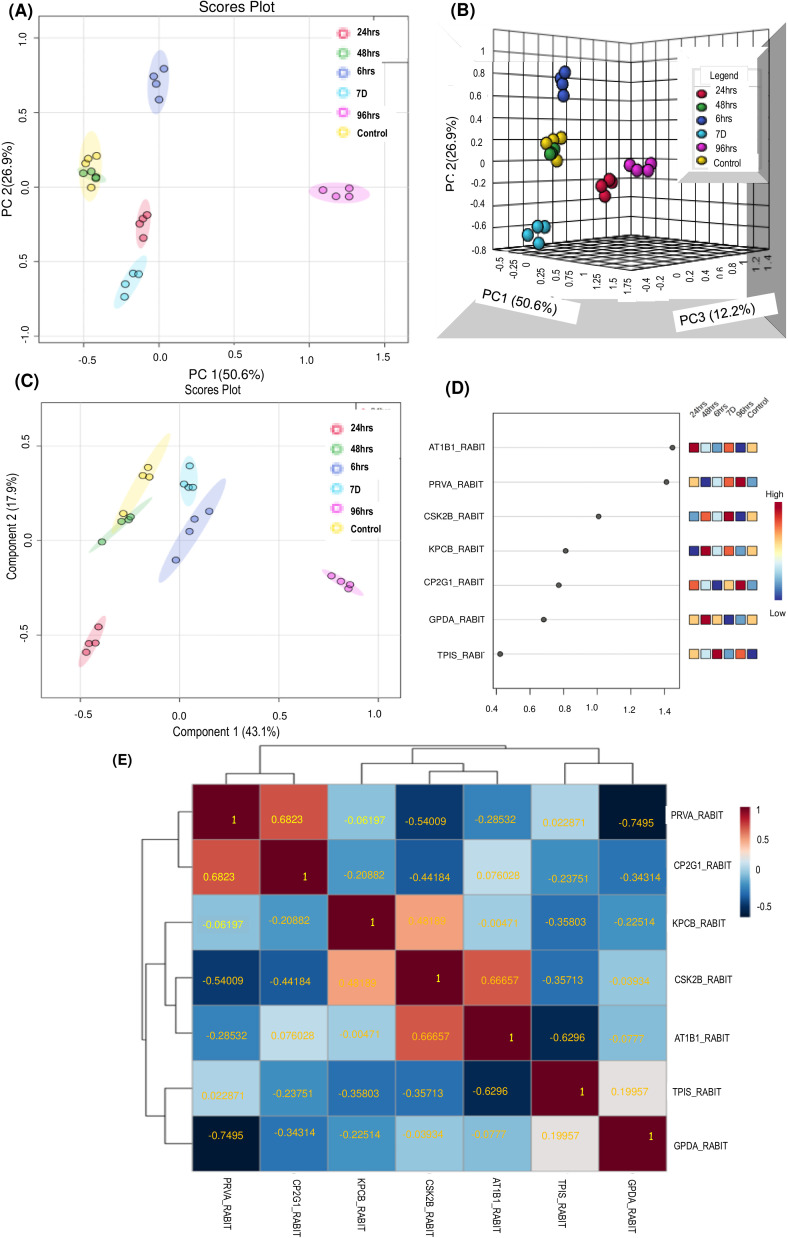
Multivariate analysis using Metaboanalyst 5.0. (A) Principal Component Analysis (PCA) - 2D PCA scores plot of the unsupervised method showing distinct clusters (B) 3D score plot between the selected PCs. (C) PLS-DA Scores plot between the selected PCs. (D) Variable Importance in Projection (VIP)-Important features identied by PLS-DA. The colored boxes on the right indicate the relative Fold Change in each group under study. (E) Graphical representation of correlation matrix. Heatmap shows correlation between differentially expressed protein spots. Each column and row defines an individual variable. Positive correlation values are in red, and negative correlation values are in blue. Hierarchical clustering was applied to both dimensions.

The dataset was investigated using Principal Component Analysis, as a multivariate unsupervised method. A PCA score plot (Fig. 7A) represents the linear combinations of the interrelated sample classes as determined by the PCs [36]. PCA data showed separation of controls from irradiated groups, however, segregation is less prominent wrt 48 h (Fig. 7A). Control, 6 h, 24 h, 48 h, 7d samples sharing the same horizontal axis (PC1, comprising the largest fraction of the sample variance, 50.6%) suggesting that protein expression pattern of the samples under these time points are somewhat similar. The 3-D score plot demonstrates that PC1, PC2, PC3 account for 50.6%, 26.9%, and 12.2% of total variation, respectively (Fig. 7B).

PCA data showed demarcation less segregation of Control from 48 h group, but PLS-DA analysis with individual irradiated group showed segregation from controls (Fig. 7C). Thus, PLS-DA may be an appropriate diagnostic model for our data as it is a better discriminator in categorizing irradiated and control groups.

Proteins able to discriminate between controls and different time points were then ordered by VIP score with Y variables (proteins) that predict X responses (samples at different time points). Differential Proteomic analysis identified seven Radiation Responsive RBC Membrane Associated Proteins (RMAPs), out of them AT1B1, PRVA, CSK2B had VIP score>1 in PLS-DA of RMAPs which were contributing to segregation in PLS-DA based on VIP score after irradiation (Fig. 7D).

Graphical representation of correlation matrix is shown in Fig. 7E signifying correlation between differentially expressed protein spots. Hierarchical clustering was applied to both dimensions. Blue indicates negative correlation, and red indicates positive correlation. Darker red indicates good correlation between the RRPs expression pattern. Correlation network exhibit clusters of Proteins with correlated change. Correlative analysis (between irradiated group) showed positive correlation between PRVA and CP2G1, KPCB and CSK2B, CSK2B and AT1B1.

Our results illustrated a Proteomic fingerprint for radiation exposure that elucidates perturbed physiological functions. The results of the present work lay a scientific foundation for developing proteomic strategies (model) for high throughput radiation bio-dosimetry for triage.

## 4 Discussion

The haematopoietic system is severely affected by radiation not only through myelosuppression, but also by disturbing the cellular integrity and functioning. Besides leucocytes which may undergo rapid depletion through myelosuppression and/or radiation-induced apoptosis, the circulating red blood cells are also known to be affected by ionizing radiation. In fact, these are highly dynamic cells with continuous interaction with extremely diverse surroundings in different tissues [37]. Biomarker studies commonly use the serum or plasma fractions of peripheral blood, and do not usually include the RBCs, despite the fact that these rather innocuous and hugely abundant cells have a fairly dynamic nature. RBCs have a flexible character involving transmembrane proteins connected to the internal cytoskeleton, providing these cells the much-needed plasticity for extensive movement into different tissues throughout their average life of 120 days. Thus, the study of RBC membrane proteins with respect to radiation injury is of immense importance to decipher the dynamic radiation response of these cells.

Plasma membrane composition of Red Blood Cells (RBCs) has been well established over the years. Recently, proteomics studies have added significantly to the knowledge of RBC composition [38,39]. There are few very comprehensive database resources of RBC membrane proteins. A comprehensive list of red cell membrane proteins based on Mass Spectroscopy has been compiled by D'Alessandro et al. 2010 [40]. Similarly, the RBC protein database by Goodman et al. 2013 [41] contains entry of 687 potential membrane proteins, with 85 unique entries. A check against the recently developed two human RBC proteome databases, viz., RBCC (Red Blood Cell Collection; http://rbcc.hegelab.org/), and RESPIRE (Repository of  Enhanced  Structures of Proteins  Involved in the Red blood cell Environment; https://www.dsimb.inserm.fr/respire/) confirms that most of these proteins are also present in human RBCs [42,43]. The majority of radiation-responsive proteins detected in our study are generally involved in functions related to “Immune response-activating cell surface receptor signalingpathway,” “Immune response-activating signal transduction,” “Immune response-regulating signalingpathway,” and “Cell surface receptor signalingpathway,” “Protein kinaseC activity,” “Enzyme binding,” “Transition metal ion binding,” “Metal ion binding,” and “Cationbinding” etc. Especially the kinases detected herein are active participants in the cellular signaling (e.g., CSNK2B, PRKCB), and hence expected to be involved in radiation-induced intracellular molecular cascades. Certain proteins found in our study are involved in ion transport and modulations in the shape of RBCs (e.g., ATP1B1), and other candidates responding to ionizing radiation participate in the metabolic and related activities (e.g.,TPI1, GPD1 and CYP2G1). In overall protein profile that we have obtained, these various proteins seem to respond differently in terms of time kinetics after radiation exposure; while some were down regulated at 6 h (PVALB, PRKCB, GPD1,CP2G1,CSNK2B), proteins disappeared at various time points (ATP1B1), protein upregulated throughout all time points (TPI1), or showed a major cyclic response (PRKCB, ATP1B1, GPD1, CSNK2B).

The radiation-responsive PV-α detected in this study is primarily an intracellular Ca^2+^-binding protein with varied functional roles. Identified initially in Amphibian and Piscean muscle cells [44], it is part of an important family of proteins involved in regulating Ca^2+^ switching inside the cells [45,35]. While its prevalence in cardiomyocytes, neurons and few other cell types has been well established [46,34], there is no reported evidence of its presence in erythrocytes till date. The isoelectric point of PVs (α and β) varies between 4.1 and 5.2, with molecular weight ranging 10 kDa to 12.5 kDa [35]. Interestingly, the PV-α is known in varied tissues, and has slightly higher isoelectric point than 5.0 that incidentally matches the radiation-responsive protein spot detected in our study. Whether this protein is residing inside or translocating to RBCs in the event of any stress, warrants further detailed investigation. As we intend to further establish the prevalence of PV-α in circulating RBCs, the present results may only be treated as an indication.

Biological processes related to ‘Immune response-activating cell surface receptor signaling pathway’, ‘Immune response-activating signal transduction’, ‘Immune response-regulating signaling pathway’ are the three most enriched functional categories in our study. The gene enriched in this category was PRKCB (Table S2). Cellular process and Metabolic process were two of the most enriched functional categories in this study. The genes enriched in this category were PRKCB, CSNK2B, TPI1, ATP1B1, CYP2G1 (Table S2).

Erythrocyte membranes contain multiple protein kinases as reported earlier and RBC membrane phospholipid scrambling is mediated by PKC [15]. An important role for Casein Kinase has been suggested in the regulation of ribosome activity in the reticulocyte system in addition to performing some other function in matured erythrocytes [26]. The erythrocyte TPI is known to play significant role in the glycolytic metabolism as well as pathogenesis in certain disease conditions such as well-established red cell enzymopathies [30,31]. Sodium/potassium-transporting ATPase subunit beta-1 is responsible for maintenance of RBC deformability [17]. Cytochrome P450 enzymes are of immense importance as their activity can cause oxidative stress [18].

Metal ion binding is another enriched functional category under Molecular Function here and CYP2G1, PVALB, PRKCB, CSNK2B are the Proteins under this category in this study (Table S2). As DNA is the site of radiation damage it was much earlier shown that DNA damage response is influenced by metal ion binding [47]. Thus, the enriched categories under GO molecular functions in our study are also having connection to radiation responsiveness.

To further understand biological interpretation, molecular pathways revealed by KEGG analysis tool of online package http://bioinformatics.sdstate.edu/go61/ were examined for their linkages to radiation response through manual referencing in PubMed. It revealed several core pathways in our study related to cellular radiation response, oxidative damage, DNA repair, apoptosis, immune response and cell signaling.

The inBio Discover™ results (Fig. 6) demonstrated that the CSNK2B that integrates and controls multiple signaling pathways, emerged as one of the hub protein in the network. CSNK2B can perform its functions by interacting with EIF3B, BRF1, HEXIM2, UTP14A, BRDT, CSNK2A1, CSNK2A2 etc. Emerging evidence suggests that CSNK2B genes encode the DNA binding subunit of the NF-κB protein complex and the beta subunit of casein kinase II (CK2) and are involved in the activation of the NF-B pathway [48]. Protein Kinases are responsible for radiation resistance and/or sensitivity [49] and one of the calcium and diacylglycerol (DAG) dependent kinase PRKCB [50] was another important hub protein in our study. Protein phosphorylation is a post-translational alteration that influences the transmission of biological signals. Protein kinases catalyze the transfer of phosphate groups to serine, threonine, and tyrosine residues on protein substrates, therefore inducing phosphorylation [49]. inBio discover™ results showed that PRKCB was found to interact with PRKCD, PDPK1, GRK2, H1–0, PHLPP2, TOP2A etc. ATP1B1 is another top protein in the network in our study. ATP1B1 was found to interact with ATP1A1, ATP1A3, FXYD2, ATP1B2 etc.

## 5 Concluding remarks

For rapid and high throughput triage of radiation incidents, the development and confirmation of early-response radiation damage biomarkers is crucial. It is necessary to have biomarkers that can be recognised at an early time point in an uncomplicated and speedy manner with the highest feasible degree of precision. Proteomic tools have long been used for understanding the radiation responses and detect biomarkers from peripheral blood or normal tissues [51]. However, there is no study on RBC membrane so far done in this regard. In the present study, we propose that radiation biomarkers from RBC membrane proteins can be used as a favoured approach. First-time, in vivo identification of radiation-induced candidate protein signatures from rabbit RBC membrane, and development of a candidate protein panel that could be used for rapid triage up to seven days after radiation exposure, are the outcome of this study. In due course in future, candidate proteins will be validated with targeted proteomics to develop signatures and radiation biomarkers. Although the identified Radiation Responsive RBC Membrane Associated Proteins (RMAPs) need to be corroborated using functional assays, we were able to show that the candidate biomarkers are involved in core pathways related to DNA damage response, cellular radiation response, oxidative damage, DNA repair, apoptosis, immune response and cell signaling. The candidate protein biomarker panel must be optimized and validated further in order to serve as an appropriate tool for advanced high-throughput screening methods through rapid immunodetection of Radiation Responsive RBC Membrane Associated Protein (RMAPs) biomarkers in blood samples for radiological emergency triage.

## Significance

Following a radiological/nuclear incident, rapid triage through biological dosimetry assays is an utmost necessity for handling of major radiation emergencies. Besides leucocytes which may undergo rapid depletion through myelosuppression and/or radiation-induced apoptosis, the circulating red blood cells are also known to be affected by ionizing radiation. RBCs have a flexible character involving transmembrane proteins connected to the internal cytoskeleton, providing these cells the much-needed plasticity for extensive movement into different tissues. Thus, the study of RBC membrane proteins with respect to radiation injury is of immense importance to decipher the dynamic radiation response of these cells. However, there is no study conducted on the effect of ionizing radiation on protein profile of erythrocytes membrane associated proteins, despite the abundance of this cell type in the blood. In the present study, we have identified differentially expressed proteins associated with RBC ghosts/membranes at early as well as late time-points (from 6h up to 7 days) following whole-body γ-irradiation of rabbits at a clinically relevant dose of 2 Gy. Bioinformatic analysis was used to identify functional associations/ enriched pathways of these candidate proteins. The results of the present work lay a scientific foundation for developing RBC based proteomic strategies for high throughput radiation bio-dosimetry for triage.

## Notes on contributors

Jubilee Purkayastha performed the experiments, analyzed the data, and wrote the manuscript.

Sudhir Chandna conceptualized the idea and designed the study.

Priyanka Grover, Kamendra Kumar, Prabuddho Mukherjee participated in executing the experiments.

## Funding

This work was supported by the grant from DRDO, Ministry of defence, India (DRDO Project No. TD-15/INM-313).

## Declaration of Competing Interest

The authors report no conflicts of interest. The authors alone are responsible for the content and writing the paper.

## Data Availability

The data that has been used is confidential.

## Appendix Supplementary materials

Image, application 1

Image, application 2

Image, application 3
