# Supplementary material for: Identification of radiation responsive RBC membrane associated proteins (RMAPs) in whole-body γ-irradiated New Zealand white rabbits
Source: Biotechnol Rep (Amst). 2023 Jan 18;37:e00783. doi: 10.1016/j.btre.2023.e00783 (PMC9883204; doi:10.1016/j.btre.2023.e00783)
Supplement: Supplementary file 1 [file mmc1.docx]

| **Table S1. Homologous proteins of the Differential Protein spots identified from Radiation Responsive RBC Membrane Associated Proteins (RMAPs) of Whole Body γ-irradiated New Zealand White Rabbits (Radiation Dose: 2 Gy). The table reports homologous proteins with: 1) Percent identity, 2) Max score (%), 3) E-value.** | | | | | |
| --- | --- | --- | --- | --- | --- |
| **Spot No.** | **Protein Identity** | **Closest homologs** | **Identity (%)** | **Max score (%)** | **E-Value** |
| 1 | Parvalbumin alpha OS=Oryctolagus cuniculus OX=9986 GN=PVALB PE=1 SV=2  (ID: PRVA_RABIT; AC: P02624) | Parvalbumin alpha  [Callithrix jacchus] | 90.00 | 168 | 1e-51 |
|  |  | Parvalbumin alpha [Homo sapiens] | 87.27 | 192 | 4e-64 |
|  |  | Parvalbumin alpha [Mus musculus] | 84.55 | 183 | 2e-60 |
| 3 | Protein kinase C beta type OS=Oryctolagus cuniculus OX=9986 GN=PRKCB PE=2 SV=3  (ID: KPCB_RABIT, AC: P05772; P05773;) | Protein kinase C beta type isoform X2 [Otolemur garnettii] | 99.55 | 1398 | 0.0 |
|  |  | Protein kinase C beta type isoform X2  [Perognathus longimembris pacificus | 99.40 | 1397 | 0.0 |
|  |  | Protein kinase C beta type isoform X2  [Sus scrofa] | 99.40 | 1397 | 0.0 |
| 5 | Casein kinase II subunit beta OS=Oryctolagus cuniculus OX=9986 GN=CSNK2B PE=2 SV=1  (ID: CSK2B_RABIT, AC: P67873; P07312; P13862) | Casein kinase II subunit beta isoform X1  [Pogona vitticeps] | 100 | 422 | 2e-148 |
|  |  | Casein kinase II subunit beta isoform X1  [Ictidomys tridecemlineatus] | 100 | 422 | 2e-148 |
|  |  | Casein kinase II subunit beta  [Varanus komodoensis] | 100 | 422 | 4e-121 |
| 6 | Triosephosphate isomerase OS=Oryctolagus cuniculus OX=9986 GN=TPI1 PE=1  SV=1  (ID: TPIS_RABIT, AC: P00939) | PREDICTED: triosephosphate isomerase  [Panthera pardus] | 99.60 | 511 | 0.0 |
|  |  | Triosephosphate isomerase  [Acinonyx jubatus] | 99.60 | 510 | 0.0 |
|  |  | Triosephosphate isomerase  [Felis catus] | 99.60 | 510 | 0.0 |
| 12a | Sodium/potassium-transporting ATPase subunit beta-1 OS=Oryctolagus cuniculus  OX=9986 GN=ATP1B1 PE=1 SV=1  (ID: AT1B1_RABIT, AC: Q9TT37) | Sodium/potassium-transporting ATPase subunit beta-1  [Ochotona curzoniae] | 94.06 | 563 | 0.0 |
|  |  | PREDICTED: sodium/potassium-transporting ATPase subunit beta-1 [Propithecus coquereli] | 92.41 | 552 | 0.0 |
|  |  | PREDICTED: sodium/potassium-transporting ATPase subunit beta-1 [Cercocebus atys] | 91.75 | 551 | 0.0 |
| 13a | Glycerol-3-phosphate dehydrogenase [NAD(+)], cytoplasmic OS=Oryctolagus  cuniculus OX=9986 GN=GPD1 PE=3 SV=2  (ID: GPDA_RABIT, AC: P08507) | PREDICTED: glycerol-3-phosphate dehydrogenase [NAD(+)], cytoplasmic [Rhinopithecus bieti] | 93.70 | 634 | 0.0 |
|  |  | Glycerol-3-phosphate dehydrogenase [NAD(+)], cytoplasmic [Rhinopithecus roxellana] | 93.98 | 634 | 0.0 |
|  |  | Glycerol-3-phosphate dehydrogenase [NAD(+)], cytoplasmic [Callithrix jacchus] | 93.70 | 633 | 0.0 |
| 18 | Cytochrome P450 2G1 OS=Oryctolagus cuniculus OX=9986 GN=CYP2G1 PE=1 SV=1  (ID: CP2G1_RABIT, AC: P24461) | Cytochrome P450 2G1  [Ochotona princeps] | 94.74 | 883 | 0.0 |
|  |  | Cytochrome P450 2G1  [Ochotona curzoniae] | 94.53 | 907 | 0.0 |
|  |  | Cytochrome P450 2G1  [Tupaia chinensis] | 92.51 | 890 | 0.0 |
