# Supplementary material for: Identification of radiation responsive RBC membrane associated proteins (RMAPs) in whole-body γ-irradiated New Zealand white rabbits
Source: Biotechnol Rep (Amst). 2023 Jan 18;37:e00783. doi: 10.1016/j.btre.2023.e00783 (PMC9883204; doi:10.1016/j.btre.2023.e00783)
Supplement: Supplementary file 2 [file mmc2.docx]

**Table S2: The top 10 Enriched GO/Pathways of the Radiation Responsive RBC Membrane Associated Proteins (RMAPs) with Fold Enrichment, FDR and the Genes/Proteins Involved under GO ‘Biological Processes’, ‘Molecular Function’, ‘Cellular Components’ as revealed by using the online package http://bioinformatics.sdstate.edu/go/.**

| **GO ‘Biological Processes’** | | | | | | |
| --- | --- | --- | --- | --- | --- | --- |
| **Enrichment FDR** | **nGenes** | **Pathway Genes** | **Fold Enrichment** | **Enriched GO /Pathways** | **URL with GO Term** | **Genes/ Proteins involved from the 07 RRPs** |
| 8.60E-03 | 1 | 5 | 643.0333333 | Immune response-activating cell surface receptor signaling pathway | http://amigo.geneontology.org/amigo/term/GO:0002429 | PRKCB |
| 8.60E-03 | 1 | 5 | 643.0333333 | Immune response-activating signal transduction | http://amigo.geneontology.org/amigo/term/GO:0002757 | PRKCB |
| 8.60E-03 | 1 | 5 | 643.0333333 | Immune response-regulating signaling pathway | http://amigo.geneontology.org/amigo/term/GO:0002764 | PRKCB |
| 5.49E-03 | 2 | 53 | 121.327044 | Phosphorylation | http://amigo.geneontology.org/amigo/term/GO:0016310 | PRKCB TPI1 |
| 5.49E-03 | 2 | 57 | 112.8128655 | Cell surface receptor signaling pathway | http://amigo.geneontology.org/amigo/term/GO:0007166 | PRKCB CSNK2B |
| 6.99E-03 | 2 | 76 | 84.60964912 | Phosphorus metabolic process | http://amigo.geneontology.org/amigo/term/GO:0006793 | PRKCB TPI1 |
| 6.99E-03 | 2 | 76 | 84.60964912 | Phosphate-containing compound metabolic process | http://amigo.geneontology.org/amigo/term/GO:0006796 | PRKCB TPI1 |
| 5.49E-03 | 3 | 283 | 34.08303887 | Response to stimulus | http://amigo.geneontology.org/amigo/term/GO:0050896 | CYP2G1 PRKCB CSNK2B |
| 5.49E-03 | 3 | 322 | 29.95496894 | Metabolic process | http://amigo.geneontology.org/amigo/term/GO:0008152 | CYP2G1 PRKCB TPI1 |
| 1.83E-03 | 4 | 537 | 23.94909994 | Cellular process | http://amigo.geneontology.org/amigo/term/GO:0009987 | PRKCB CSNK2B TPI1 ATP1B1 |
|  |  |  |  |  |  |  |
| **GO ‘ Molecular Function’** | | | | | | |

| **Enrichment FDR** | **nGenes** | **Pathway Genes** | **Fold Enrichment** | **Enriched GO /Pathways** | **URL with GO Term** | **Genes/ Proteins involved from the 07 RRPs** |
| --- | --- | --- | --- | --- | --- | --- |
| 4.45E-03 | 1 | 5 | 643.0333333 | Protein kinase c activity | http://amigo.geneontology.org/amigo/term/GO:0004697 | PRKCB |
| 4.73E-04 | 2 | 34 | 189.127451 | Enzyme binding | http://amigo.geneontology.org/amigo/term/GO:0019899 | TPI1 ATP1B1 |
| 1.94E-03 | 2 | 79 | 81.39662447 | Transition metal ion binding | http://amigo.geneontology.org/amigo/term/GO:0046914 | CYP2G1 PRKCB |
| 3.91E-06 | 4 | 202 | 63.66666667 | Metal ion binding | http://amigo.geneontology.org/amigo/term/GO:0046872 | CYP2G1 PVALB PRKCB CSNK2B |
| 3.91E-06 | 4 | 206 | 62.43042071 | Cation binding | http://amigo.geneontology.org/amigo/term/GO:0043169 | CYP2G1 PVALB PRKCB CSNK2B |
| 3.71E-03 | 2 | 116 | 55.43390805 | Molecular function regulator | http://amigo.geneontology.org/amigo/term/GO:0098772 | PRKCB CSNK2B |
| 2.58E-04 | 3 | 197 | 48.96192893 | Protein binding | http://amigo.geneontology.org/amigo/term/GO:0005515 | PRKCB TPI1 ATP1B1 |
| 1.36E-05 | 4 | 302 | 42.58498896 | Ion binding | http://amigo.geneontology.org/amigo/term/GO:0043167 | CYP2G1 PVALB PRKCB CSNK2B |
| 1.40E-08 | 6 | 477 | 40.44234801 | Binding | http://amigo.geneontology.org/amigo/term/GO:0005488 | CYP2G1 PVALB PRKCB CSNK2B TPI1 ATP1B1 |
| 4.94E-04 | 3 | 274 | 35.20255474 | Catalytic activity | http://amigo.geneontology.org/amigo/term/GO:0003824 | CYP2G1 PRKCB TPI1 |
| **GO ‘Cellular Components’** | | | | | | |
| **Enrichment FDR** | **nGenes** | **Pathway Genes** | **Fold Enrichment** | **Enriched GO /Pathways** | **URL with GO Term** | **Genes/ Proteins involved from the 07 RRPs** |
| 6.22E-03 | 1 | 3 | 1071.722222 | Sodium:potassium-exchanging atpase complex | http://amigo.geneontology.org/amigo/term/GO:0005890 | ATP1B1 |
| 6.22E-03 | 1 | 5 | 643.0333333 | Cation-transporting atpase complex | http://amigo.geneontology.org/amigo/term/GO:0090533 | ATP1B1 |
| 6.22E-03 | 1 | 5 | 643.0333333 | ATPase dependent transmembrane transport complex | http://amigo.geneontology.org/amigo/term/GO:0098533 | ATP1B1 |
| 6.22E-03 | 2 | 170 | 37.8254902 | Protein-containing complex | http://amigo.geneontology.org/amigo/term/GO:0032991 | CSNK2B ATP1B1 |
| 1.61E-03 | 3 | 370 | 26.06891892 | Membrane | http://amigo.geneontology.org/amigo/term/GO:0016020 | CYP2G1 PRKCB ATP1B1 |
| 1.61E-03 | 3 | 403 | 23.93424318 | Intracellular | http://amigo.geneontology.org/amigo/term/GO:0005622 | PRKCB CSNK2B TPI1 |
| 1.19E-02 | 2 | 297 | 21.65095398 | Membrane-bounded organelle | http://amigo.geneontology.org/amigo/term/GO:0043227 | CYP2G1 PRKCB |
| 1.38E-02 | 2 | 353 | 18.21624174 | Organelle | http://amigo.geneontology.org/amigo/term/GO:0043226 | CYP2G1 PRKCB |
| 1.38E-02 | 2 | 359 | 17.91179201 | Cytoplasm | http://amigo.geneontology.org/amigo/term/GO:0005737 | PRKCB TPI1 |
| 7.87E-04 | 4 | 726 | 17.7144169 | Cellular anatomical entity | http://amigo.geneontology.org/amigo/term/GO:0110165 | CYP2G1 PRKCB TPI1 ATP1B1 |
