# Supplementary figures and images for: Identification of radiation responsive RBC membrane associated proteins (RMAPs) in whole-body γ-irradiated New Zealand white rabbits
Source: Biotechnol Rep (Amst). 2023 Jan 18;37:e00783. doi: 10.1016/j.btre.2023.e00783 (PMC9883204; doi:10.1016/j.btre.2023.e00783)

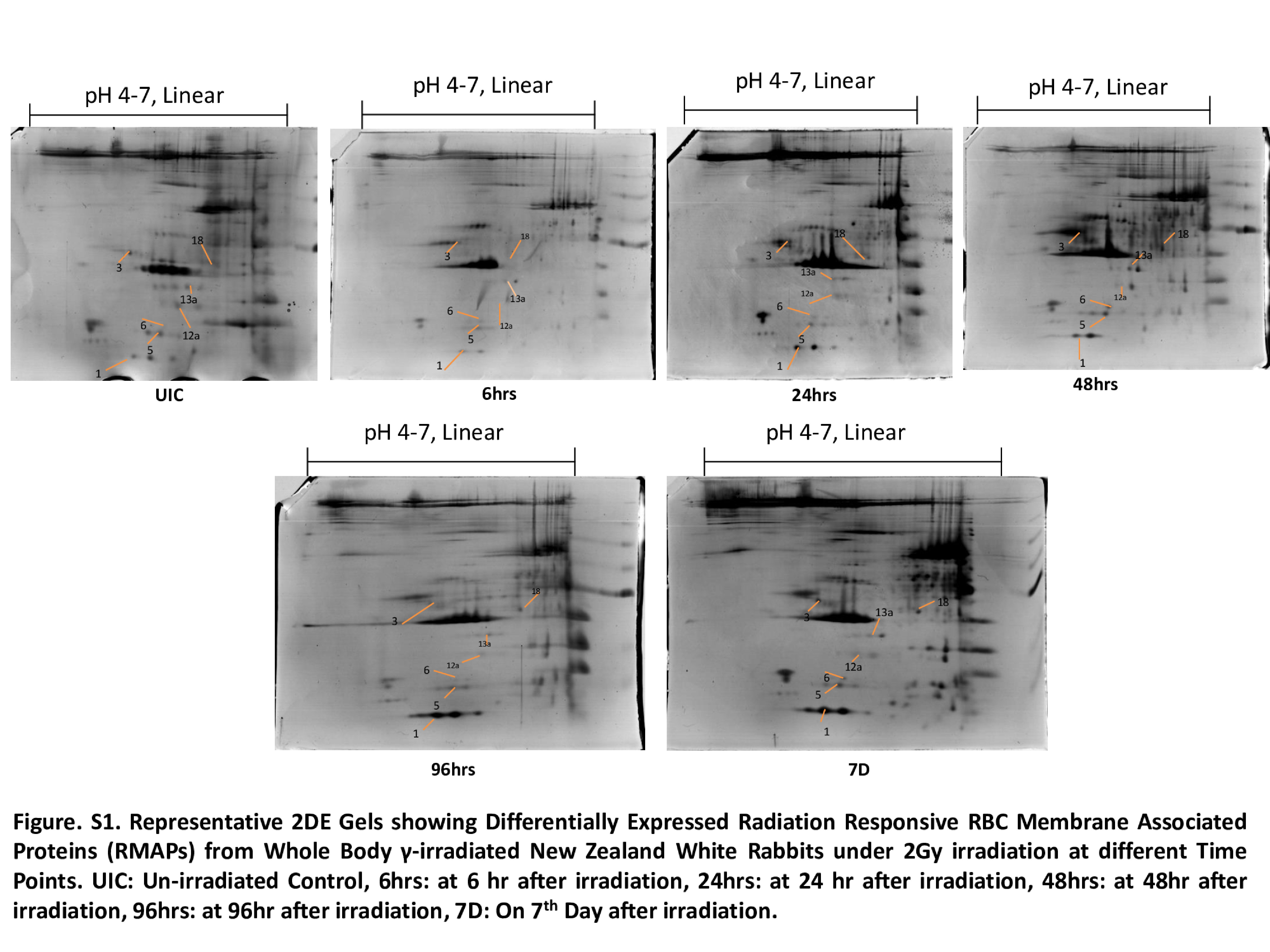

Supplement: Supplementary file 3 [file mmc3.jpg]
